# Impact of a narrative medicine program on reflective capacity and empathy of medical students in Iran

**DOI:** 10.3352/jeehp.2020.17.3

**Published:** 2020-01-27

**Authors:** Saeideh Daryazadeh, Payman Adibi, Nikoo Yamani, Roya Mollabashi

**Affiliations:** 1 Department of Medical Education, Medical Education Research Center, Isfahan University of Medical Sciences, Isfahan, Iran; 2 Department of Internal Medicine, Research Center of Gastroenterology, Isfahan University of Medical Sciences, Isfahan, Iran; 3 Medical Education Research Center, Isfahan University of Medical Sciences, Isfahan, Iran; Hallym University, Korea

**Keywords:** Communication, Empathy, Iran, Medical students, Narrative medicine

## Abstract

**Purpose:**

Narrative medicine consists of the expression of medical experiences and the reflection on narratives to foster empathic communication with patients. Reflecting on narratives increases self-awareness and recognition of the feelings of the narrator or the story’s main character, which in turn affects the audience. This study was conducted to examine the impact of a narrative medicine program on the reflective capacity and empathy of medical students.

**Methods:**

A quasi-experimental study was performed during the 2018–2019 academic year at Isfahan University of Medical Sciences in Iran involving 135 medical interns in 2 groups (control [n=66] and experimental [n=69]). Interns in the experimental group took part in seven 2-hour reflective practice sessions, while those in the control group underwent no educational intervention. Pre-test and post-test assessments were conducted for both groups using 2 valid and reliable tools for the assessment of reflective capacity and empathy. Mean reflection and empathy scores were compared within groups (between pre- and post-test values) and between groups (using the paired-t test and the t-test; P≤0.05).

**Results:**

The mean reflection and empathy scores of the experimental group significantly increased from pre-test to post-test, but those of the control group did not. Moreover, the mean post-test scores were significantly different between the 2 groups (P<0.001).

**Conclusion:**

Narrative medicine is an effective teaching method that can improve reflective capacity and empathy, thereby ultimately promoting professionalism as a core competency in medicine. Consideration of learning conditions and interdisciplinary teaching are necessary for implementing a narrative medicine program.

## Introduction

### Background

Since the 1990s, the use of narrative in medicine has increased with the goal of improving reflection and self-awareness, and the impact of this change on professionalism and patient care has been emphasized. The narrative approach involves writing stories about clinical experiences and patients [1]. The term “narrative medicine” (NM) was used for the first time in 2000, when it was described by Charon et al. [1] as a model for effective practice in the medical humanities [2]. The definition of NM, in the words of Charon, is “clinical practice fortified with the narrative competence to recognize, absorb, interpret, and honor the stories of self and other” [3]. In NM, patient-centered and relationship-based approaches are emphasized, along with humanism, empathy, interpersonal communication, and respect for patients [1].

As such, medical science does not merely comprise history-taking, physical examination, diagnosis, and treatment. Instead, a physician using the narrative approach must engage deeply in patient treatment [2], which can facilitate professional and personal development and improve self-reflection and empathy [4]. Reflection is an important competency in the health care system [5]. Reflective capacity refers to reflecting about the experiences of oneself and others in a way that influences future behavior; from the viewpoint of Mann and colleagues, it involves “critical analysis of knowledge and experience to achieve deeper meaning and understanding” [5,6]. Charon described the reflective exercise as a physician’s experience of and reflections on encounters with patients, which are associated with the expansion of emotional capacity through the improvement of empathy and patient care [7].

Empathy in the context of health care is defined as “a cognitive attribute that involves an ability to understand the patient’s inner experiences and perspective and a capability to communicate this understanding.” Empathy facilitates patient satisfaction, acceptance, and proper follow-up, along with the achievement of better therapeutic outcomes and the reduction of patient complaints resulting from physicians’ decision-making and medical errors [8]. Studies have shown that empathy decreases during medical school. However, educational interventions may improve empathy in medical students [8].

### Purpose

Previously, we lacked methods for teaching or assessing the reflective capacity and empathy of medical students at our university. This study was conducted to examine the impact of a NM program on the reflective capacity and empathy of medical students. The specific goal was to measure the mean pre-test and post-test scores of reflection and empathy of students in the control and experimental groups and to compare those scores within and between groups.

## Methods

### Ethics statement

This research was part of a project with the ethics code IR.MUI.REC.1396.3.472 at Isfahan University of Medical Sciences in Iran. We informed participants about the educational objectives of the study and communicated to them that their information was only available to the research team. Informed consent was obtained from the subjects.

### Study design

This quasi-experimental study was performed during the 2018–2019 academic year with 2 control and experimental groups under a nonequivalent group design. Pre-test and post-test evaluations were performed in both groups using 2 tools, which are described below. We obtained permission from the tool developers to use them in our study and utilized articles about those tools as references.

### Participants

A total of 135 medical interns participated in this study during their 3-month internal medicine internship at the medical school. The control group included 66 medical interns who did not undergo the educational intervention, while the experimental group included 69 medical interns who participated in the NM program. Interns were randomly assigned to a 3-month internship in the internal medicine section by the supervisor in charge. We selected 2 consecutive groups of medical interns, the first of which was the control group and the second the experimental group. We utilized this sequence in order to avoid disclosure of information about the educational intervention among interns.

To test the difference between 2 independent means, an adequate sample size was estimated to be 122 with the following input parameters according to Cohen’s power analysis: effect size (D), 0.6; α error probability, 0.05; power (1-β probability), 0.95; and allocation ratio (N2/N1), 1 [9].

### Tools

#### The modified Reflection Evaluation for Learners’ Enhanced Competencies Tool rubric: a tool to assess reflection

We chose the Reflection Evaluation for Learners’ Enhanced Competencies Tool (REFLECT) rubric, developed by Wald et al. [6], to teach and assess reflection in medical interns. This tool introduces 4 levels of reflection (non-reflective; habitual action, non-reflective; thoughtful action, reflective; and critically reflective) and 5 dimensions (writing spectrum, presence, description of conflict or disorienting dilemma, attending to emotions, and analysis and meaning-making). The validity, reliability, and feasibility of this tool have been verified (intraclass correlation coefficient [ICC], 0.632; Cronbach α, 0.774). The ease of use and inter-rater reliability of the REFLECT rubric have also been confirmed, and the tool is recommended for the teaching of reflection in medical schools [6]. We used a modified Persian version of the REFLECT system for quantitative pre-test and post-test assessments. In this version, numerical values correspond to reflection levels ranging from 1 (habitual action) to 4 (critical reflection). The total score obtained using this tool ranges from a minimum of 5 to a maximum of 20 [10] (Supplement 1).

#### The Jefferson Scale of Physician Empathy: a tool to assess empathy

Previous studies have used various tools to assess empathy. We chose the Jefferson Scale of Physician Empathy (JSPE), developed by Hojat and colleagues, which is a reliable, valid self-report tool and the most well-known of related tools. This tool comprises 20 items rated on a 7-point Likert scale ranging from 7 (strongly agree) to 1 (strongly disagree) [8]. We used a valid and reliable Persian version of the JSPE, as confirmed in a study by Hashemipour and Karami [11], with a 5-point Likert scale (ICC, 0.82; Cronbach α, 0.83). The total score obtained using this tool varies from 20 to 100 (Supplement 2).

### Validity and reliability of tools

The face and content validity of both tools have been confirmed in past studies, and we measured the reliability of the tools by assessing internal consistency through the Cronbach α coefficient.

### Inter-rater reliability in the REFLECT rubric

We chose 2 raters to assess the reflective narratives of the medical interns. One of the raters was a medical education specialist who worked in NM, and the other rater was a narrative analyst who was also a physician. The 2 raters independently assessed 30 reflective narratives of interns using the modified REFLECT rubric, and we then measured the inter-rater agreement using the Cohen kappa coefficient.

### Setting

#### The narrative medicine program

We used the framework of a NM program based on Gagne’s instructional design model to train the medical interns [12]. This framework focuses on improving educational effectiveness, learning, and small-group reflective practice. The program incorporates the 3-stage model of NM presented by Charon et al. [1], where the 3 stages are reading, reflecting, and group discussion.

#### Pre-test

Prior to the initiation of training sessions, we conducted a pre-test evaluation in which we asked the students to write a reflective narrative about the use of empathy with a patient. The narratives were assessed by 2 trained raters using the REFLECT rubric. We also used the JSPE questionnaire tool to measure students’ empathy scores.

#### Educational sessions

For the purpose of reflective practice, we divided the interns into small groups of 8–12. The NM program consisted of seven 2-hour sessions in 2 parts (theoretical and practical) that medical interns practiced in small groups, with a focus on reflective narrative writing. A clinical educator was responsible for directing each small group of medical interns, and a narrative analyst rotated between the small groups.

#### Post-test

After the conclusion of the sessions, we administered a post-test evaluation, in which we asked students to complete the JSPE tool and to provide a reflective narrative based on the lessons they had learned. The raters again assessed the student narratives based on the 4 levels of reflection (from 1 to 4) and the 5 dimensions specified in the modified REFLECT rubric.

### Data analysis

The mean scores of students on the pre-test and post-test within groups and between groups (control and experimental) were compared using parametric analysis, the paired-t test, and the t-test (P≤0.05). Data analysis was performed using IBM SPSS software ver. 23.0 (IBM Corp., Armonk, NY, USA) (Dataset 1).

## Results

The participants in the control group comprised 66 students ranging from 22 to 27 years old, with a mean age of 24.77±1.05 years. The experimental group consisted of 69 students ranging from 23 to 28 years old, with a mean age of 24.96±0.96 years (Table 1).

### Reliability of the tools

The internal consistency of the modified REFLECT and JSPE tools was measured to confirm their reliability. The Cronbach α coefficients of these tools were 0.83 and 0.82, respectively (N=66).

### Inter-rater reliability

The kappa agreement coefficient was 0.779, with a significance of 0.000. In the assessment of reflection, this constituted a good agreement between the raters (n=30).

### Reflection

Comparisons of the mean reflection scores in the pre-test and post-test evaluations of each group are shown in Table 2. The mean reflection score across all dimensions significantly increased in the experimental group after the NM program (P<0.001). In the control group, however, no significant difference was observed. Moreover, the pre-test values of the mean reflection score in the 2 study groups did not significantly differ across all dimensions, but the difference between the post-test results was significant (P<0.001).

### Empathy

The pre-test and post-test mean empathy scores were also compared in both groups. The mean empathy score in the experimental group significantly increased after NM compared to the pre-test value. However, in the control group, no significant difference between the pre- and post-test scores was found. Furthermore, the mean pre-test empathy scores did not significantly differ between the 2 groups, but the mean post-test empathy scores did show a significant difference (P<0.001) (Table 3). In brief, the mean reflection and empathy scores increased in the post-test results of the experimental group.

## Discussion

### Key results

This study examined the impact of an NM program on reflection and empathy in medical students. The results showed that the NM program improved reflection and ultimately led to increased empathy.

A comparison of mean pre-test and post-test reflection scores in the 2 groups showed that the reflection score improved in the experimental group along all dimensions of a reflective narrative. In addition, the dimensions of critical reflection and analysis and meaning-making were not observed in the pre-test results of either group or in the post-test results of the control group, but they were observed in the post-test results of the experimental group. In this study, the 2 groups were designed to facilitate the accurate analysis of the program’s impact, which could serve as a basis for more in-depth studies examining other similar factors. As evidenced by the results of this study, the impact of the NM program on students’ empathy through reflective practice is indisputable. These results confirm the need for formal education in this field.

### Comparison with other studies

Previous studies have assessed the impacts of educational interventions (such as illness narratives) and art (such as poetry, storytelling, reflective practice, and film) in the development of empathy. These investigations have examined the improvement of doctor-patient relationships and the maturation of the affective dimension of empathy. The results show that empathy improves after NM intervention [8,13-15]. The results of these studies—namely, that reflective practice improves empathy—are consistent with the results of the present study. However, not all NM intervention has produced positive outcomes with regard to empathy. One study reported that 1 year after the end of NM seminars, there was only a small increase in participants’ empathy scores, and burnout continued to be high [4]. Hence, it is important to conduct ongoing training and qualitative studies to explore the effects of other factors on empathy, and we cannot say for certain that NM will lead to empathetic interactions with patients in future clinical practice [4]. It is therefore necessary to examine the long-term outcomes of NM programs in future studies.

In addition, at Columbia University, weekly seminars are held in which student narratives are read from all medical departments that conduct narrative-related programs [1]. These seminars can introduce students to the ethical challenges of different educational departments and facilitate discussion. The potential benefits of interdisciplinary narrative seminars include friendly interactions between faculty and students, fostering empathy with patients, and understanding ethical challenges. It seems that presenting the NM program for undergraduate medical students, especially from the beginning of clinical education, has a greater impact on empathy improvement. However, the effectiveness of NM may also be related to its cultural acceptance by students. Because most studies related to NM interventions have been conducted in the West [2], it is useful to conduct other local surveys in this field, especially with regard to the culture of Eastern societies.

Given the importance of developing reflective capacity, some educational tools and practice guides in this area have been developed at different universities. Charon et al. [1] at Columbia University developed a reflective practice guide for clinical care and empathy with patients and provided a training tool using the contents of the NM seminars. In addition, at Brown University, Wald et al. [16] conducted a reflective writing rubric for pre-clinical students with clinical skills and professional skills training and early exposure to patients. Medical students received individual feedback from faculty members in behavioral science about reflection in their narrative writings to foster critical thinking and the affective dimension. Student assessments were carried out qualitatively, and they reported that the use of reflective practice had outcomes such as creating deeper reflective skills, providing interdisciplinary feedback, improving teamwork, and facilitating professional and individual growth [16]. We used the Persian version of the modified REFLECT rubric to practice reflective narrative [10], and we recommend using practical guides for practicing reflective writing to train students at different levels. It is also recommended to perform more studies on the impact of reflective practice on other aspects of medical education, such as ethical reasoning, critical thinking, decision-making, and clinical reasoning.

### Limitation

One limitation of this study was the implementation of an educational intervention in only 1 target group of medical students (interns). Another limitation of the study was that we administered the course only in the internal medicine internship program. However, considering the feasibility of implementation, we tried to provide the best possible conditions for the successful implementation of the course, which was the first ever NM program at our university. Therefore, this report can serve as a guide for implementing NM programs in other educational departments.

### Conclusion

The results of our study indicate that NM in medical education is effective for increasing reflection and empathy and ultimately promotes professionalism in medical students. Hence, goals in the medical humanities can be achieved through a combination of literature and medicine. Fostering reflection can improve many aspects of medical education, including professional development and patient-centered care, and this can be achieved by providing an NM program and guided feedback through interdisciplinary teaching. In order to achieve the desired results, we must consider the optimal conditions for program implementation and the priority of our educational goals.

## Figures and Tables

**Table 1.** Demographic characteristics of the participants (N=135)

Characteristic	Control group	Experimental group
Sex		
Female	41 (62.1)	33 (47.8)
Male	25 (37.9)	36 (52.2)
Marital status		
Single	49 (74.2)	50 (72.5)
Married	17 (25.8)	19 (27.5)

Values are presented as number (%).

**Table 2.** Comparison of mean scores for the dimensions of reflection

Group	Dimension^a)^	Pre-test				Post-test				Paired t-test		t-test	
		Mean±SD	Min	Max	Mode	Mean±SD	Min	Max	Mode	df	P-value	df	P-value
Control	1	1.41±0.58	1.00	3.00	1.00	1.36±0.54	1.00	3.00	1.00	65	0.182	133	0.096
	2	1.53±0.71	1.00	3.00	1.00	1.48±0.61	1.00	3.00	1.00		0.495		0.200
	3	1.50±0.64	1.00	3.00	1.00	1.47±0.56	1.00	3.00	1.00		0.658		0.187
	4	1.68±0.75	1.00	3.00	1.00	1.58±0.66	1.00	3.00	1.00		0.070		0.295
	5	1.04±0.21	1.00	2.00	1.00	1.03±0.17	1.00	2.00	1.00		0.568		0.084
	6	7.17±2.34	5.00	14.00	5.00	6.92±1.94	5.00	11.00	5.00		0.096		0.269
Experimental	1	1.56±0.50	1.00	3.00	2.00	3.32±0.65	2.00	4.00	3.00	68	0.000	133	0.000
	2	1.68±0.65	1.00	3.00	2.00	3.26±0.70	1.00	4.00	3.00		0.000		0.000
	3	1.64±0.57	1.00	3.00	2.00	3.55±0.63	2.00	4.00	4.00		0.000		0.000
	4	1.56±0.53	1.00	3.00	2.00	2.98±0.58	2.00	4.00	3.00		0.000		0.000
	5	1.13±0.34	1.00	2.00	1.00	2.64±0.62	1.00	4.00	3.00		0.000		0.000
	6	7.58±1.96	5.00	13.00	5.00	15.75±2.37	9.00	19.00	18.00		0.000		0.000

SD, standard deviation; df, degrees of freedom.

a) 1: writing spectrum; 2: presence; 3: description of conflict or disorienting dilemma; 4: attending to emotions; 5: analysis and meaning-making; 6: total score.

**Table 3.** Comparison of mean scores for empathy

Group	Pre-test				Post-test				Paired-t test		t-test	
	Mean±SD	Min	Max	Mode	Mean±SD	Min	Max	Mode	df	P-value	df	P-value
Control	75.86±8.50	57.00	96.00	70.00	76.35±7.99	52.00	95.00	76.00	65	0.125	133	0.184
Experimental	73.90±8.59	55.00	79.00	61.00	94.90±4.47	95.00	100.00	96.00	68	0.000	133	0.000

SD, standard deviation; df, degrees of freedom.

## Footnotes

**Data availability** Data files are available from Harvard Dataverse: https://doi.org/10.7910/DVN/BUGCNN Dataset 1. Raw data.

## Supplementary materials

Data files are available from https://doi.org/10.7910/DVN/BUGCNN

Supplement 1. The modified REFLECT in Persian.

Supplement 2. The Persian version of the JSPE.

Supplement 3. Audio recording of the abstract.
