# Supplementary material for: Impact of a narrative medicine program on reflective capacity and empathy of medical students in Iran
Source: J Educ Eval Health Prof. 2020 Jan 27;17:3. doi: 10.3352/jeehp.2020.17.3 (PMC7061215; doi:10.3352/jeehp.2020.17.3)
Supplement: Supplementary file 2 — Supplement 1. The modified REFLECT in Persian. [file jeehp-17-03-suppl1.pdf]

Supplementary materials: The Modified REFLECT In Persian

دستورالعمل REFLECT (ابزار ارزیابی بازاندیشی برای توانمندی های پیشرفته یادگیری)

	**سطح**				
	**نمره دهی**	**۱**	**۲**	**۳**	**۴**
**ردیف**	**معیار**	**عملکرد عادی بدون بازاندیشی**	**عملکرد متفکرانه و یا درون نگری**	**بازاندیشی**	**بازاندیشی نقادانه**
**۱**	**طیف نوشتن**	رویکرد نوشتن توصیفی سطحی (گزارش واقعه، برداشت مبهم) بدون بازاندیشی یا درون نگری	رویکرد نوشتن توصیفی دقیق و برداشتهای بدون بازاندیشی	حرکت به سمت بازاندیشی، فراتر از گزارش دادن یا نوشتن توصیفی (به عنوان مثال، تلاش برای فهمیدن، سوال کردن یا تحلیل رویداد)	اکتشاف و نقد تصورات، ارزش ها، باورها و/ یا سوگیری ها و پیامدهای عمل (حال و آینده)
**۲**	**حضور (وجود احساسات نویسنده در متن)**	نبود حضور نسبی احساسات نویسنده	حضور نسبی احساسات نویسنده	حضور نسبتا کامل و تاحد زیاد احساسات نویسنده	حضور کامل احساسات نویسنده
**۳**	**شرح تعارض یا معضل دلسرد کننده (موجب درماندگی)**	نبود هیچ توضیحی از معضل ناخوشایند، تعارض، چالش یا مورد نگرانی	نبود یا ضعف توصیف معضل، تعارض، چالش یا مورد نگرانی	شرح معضل ناخوشایند، تعارض، چالش یا مورد نگرانی	توصیف کامل معضل ناخوشایند، تعارض، چالش یا موضوع نگرانی دربرگیرنده دیدگاه های متعدد، بررسی توضیحات جایگزین و تصورات چالش برانگیز
**۴**	**توجه به احساسات**	کمبود یا نبودن شناخت یا توجه به احساسات	شناخت احساسات ولی بدون بازیابی یا توجه	شناخت، بازیابی و توجه به احساسات	شناخت، بازیابی، توجه به احساسات و به دست آوردن بینش احساسی
**۵**	**تحلیل و ساخت معنا**	بدون تحلیل یا ساخت معنا	تحلیل یا ساخت معنای کم یا ناواضح	تا حدودی تحلیل و ساخت معنا	تحلیل و ساختن معنای کامل
