# Supplementary material for: Impact of a narrative medicine program on reflective capacity and empathy of medical students in Iran
Source: J Educ Eval Health Prof. 2020 Jan 27;17:3. doi: 10.3352/jeehp.2020.17.3 (PMC7061215; doi:10.3352/jeehp.2020.17.3)
Supplement: Supplementary file 3 — Supplement 2. The Persian version of the JSPE. [file jeehp-17-03-suppl2.pdf]

Supplementary materials: Empathy scale-JSPE (Jefferson Scale of Physician Empathy) in Persian

پرسشنامه همدلی (Empathy)

ردیف	سوالات	کاملاً موافق	موافق	بی نظر	مخالف	کاملاً مخالف
۱	ارتباط شخصی قوی بین بیمار و همراهانش بر روی روش درمانی من تاثیری ندارد.					
۲	در صورتی که بیمار متوجه حس همدلی من شود احساس بهتری می کند.					
۳	برای من مشکل است تا محیط پیرامون خود را از دریچه چشم بیمارم ببینم.					
۴	داشتن زبان مشترک با بیمار و برقراری ارتباط کلامی با وی در ایجاد رابطه مثبت بین بیمار و پزشک بسیار مهم است.					
۵	به نظر من همدلی با بیمار سبب تاثیر بیشتر درمانهای پزشکی و جراحی بر روی وی می شود.					
۶	از آنجایی که انسانها با هم متفاوت می باشند برای من مشکل است که وضعیت بیماری بیمارم را درک کنم.					
۷	دانستن احساس بیمار و همراهانش تاثیر بر روند درمانی ایشان ندارد.					
۸	توجه به تجارب شخصی بیمار هیچگونه تاثیری بر روند درمانی آنها ندارد.					
۹	من در هنگام درمان بیمارانم خودم را به جای آنها می گذارم.					
۱۰	بیماران برای درک احسا سات شان ارزش قائل بوده و این امر در پا سخ به درمان آنها موثر می باشد					
۱۱	م شکلات بیمار تنها بایستیی با درمانهای پز شکی و جراحی بر طرف شود و همدلی جایگاهی در درمان بیماران ندارد.					
۱۲	پرسش از بیماران در رابطه با زندگی شخصی آنها در درک مشکلات آنها تاثیری ندارد .					
۱۳	من سعی می کنم از طریق توجه به بیمار، گوش دادن به حرفها و توجه به اعمالش آنچه را در فکر او می گذرد را متوجه شوم.					
۱۴	من متعقدم که همدلی با بیمار هیچگونه جایگاهی در روند درمان بیماران ندارد.					
۱۵	همدلی نوعی مهارت درمانی بوده و سبب محدودیت درمان نمی شود.					
۱۶	یک جز مهم در ایجاد ارتباط با بیمار، همدلی با بیمار و بستگانش می باشد.					
۱۷	من سعی می کنم شبیه بیمارانم فکرکنم، زیرا این موضوع در روند درمانی آنها موثر است.					
۱۸	من سعی می کنم تا در هنگام گرفتن تاریخچه و بررسی وضعیت سلامت بیمار هیچگونه احساس همدلی با وی نداشتم باشم.					

۱۹	من از مطالعه کتب و مراجع غیر رشته ام و کتب هنری لذت نمی برم.					
۲۰	من معتقدم که همدلی عامل مهمی در درمان های پزشکی می باشد.					
